# The Immune-Modulator Pidotimod Affects the Metabolic Profile of Exhaled Breath Condensate in Bronchiectatic Patients: A Metabolomics Pilot Study

**DOI:** 10.3389/fphar.2019.01115

**Published:** 2019-10-03

**Authors:** Maria D’Amato, Debora Paris, Antonio Molino, Paola Cuomo, Andrea Fulgione, Nunzia Sorrentino, Letizia Palomba, Mauro Maniscalco, Andrea Motta

**Affiliations:** ^1^Division of Pneumology, Department of Respiratory Diseases, University of Naples Federico II, AORN dei Colli-Monaldi Hospital, Naples, Italy; ^2^Institute of Biomolecular Chemistry, National Research Council, Pozzuoli, Italy; ^3^Department of Agriculture, University of Naples Federico II, Portici, Italy; ^4^Department of Biomolecular Sciences, University of Urbino Carlo Bo, Urbino, Italy; ^5^Pulmonary Rehabilitation Unit, ICS Maugeri SPA, IRCCS, Telese Terme, Italy

**Keywords:** biomarkers, bronchiectasis, disability, exhaled, metabolomics, NMR, outcome, rehabilitation

## Abstract

**Introduction:** Pidotimod, a synthetic dipeptide molecule with biological and immunological activities, is used to reduce the number of exacerbations or pneumonitis in patients with inflammatory diseases.

In the present study, we investigated whether Pidotimod modifies the metabolomic pathways measured in the exhaled breath condensate (EBC) of non-cystic fibrosis bronchiectatic patients (NCFB).

**Materials and Methods:** We analyzed 40 adult patients affected by NCFB. They were randomly selected to receive Pidotimod 800 mg b/d for 21 consecutive days (3 weeks) per month for 6 months (20 patients, V_1_ group) or no drug (20 patients, V_0_ group), with a 1:1 criterion and then followed as outpatients.

**Results:** EBC samples were collected from all patients at baseline and after 6 months. They were investigated by combined nuclear magnetic resonance (NMR) spectroscopy and multivariate statistical analysis to uncover metabolic differences between EBC from NCFB patients before and after therapy with Pidotimod. Pulmonary function test and pulmonary exacerbations were analyzed at baseline and at the end of Pidotimod therapy.

The EBC metabolites were all identified, and through statistical evaluation, we were able to discriminate the two samples’ classes, with acetate, acetoin, lactate, and citrate as statistically significant discriminatory metabolites. The model vas validated by using a blind set of 20 NCFB samples, not included in the primary analysis.

No differences were observed in PFT after 6 months. At the end of the study, there was a significant decrease of exacerbation rate in V_1_ group as compared with V_0_ group, with a substantial reduction of the number of mild or severe exacerbations (*p* < 0.001).

**Discussion:** Pidotimod modifies the respiratory metabolic phenotype (“metabotype”) of NCFB patients and reduces the number of exacerbations.

## Introduction

Bronchiectasis represents one of the most important healthcare problems due to the high mortality rate and the increased incidence worldwide (King et al., 2006). It is a pulmonary chronic inflammatory disease characterized by bronchial dilatation that can develop in response to many etiologies, like acquired conditions (infection, pulmonary fibrosis, recurrent, or chronic aspiration), as well as congenital conditions [cystic fibrosis (CF), primary ciliary dyskinesia (PCD)] (Chalmers et al., 2012), all leading to bronchus anomalies. Bronchiectasis is associated with chronic cough and sputum production, and, as a chronic lung disorder, it brings about poor quality of life and frequent exacerbations. The economic burden of this disease has been estimated to be similar to chronic obstructive pulmonary disease (COPD). It obviously increases with disease severity, hospitalizations, need for intensive care, and use of inhaled antibiotics (Goeminne et al., 2019). Therefore, there is a continuous search for a pharmacological therapy useful to improve patient conditions and reduce the economic burden (Kocurek and Jagana, 2019).

Pidotimod is a synthetic dipeptide molecule with biological and immunological activities on the adaptive and the innate immune responses, as suggested by *in vivo* and *in vitro* studies (Riboldi et al., 2009; Esposito et al., 2015). Pidotimod has been used to reduce exacerbations or pneumonitis in patients with chronic inflammatory disorders (Trabattoni et al., 2017).

In the last few years, metabolomics has become a leading tool in defining disease phenotype because it is able to correlate the metabolic dysregulation with the phenotype. Metabolomics is a useful tool to investigate airway diseases, their treatment, and follow-up as the respiratory tract offers a natural matrix (exhaled breath), which is easily collected as condensate (EBC) (Maniscalco et al., 2018a; Maniscalco et al., 2019). Nuclear magnetic resonance (NMR)–based metabolomics of EBC has progressively gained importance for quantitative determination of the metabolic response to several respiratory disorders (Paris et al., 2018). It unambiguously recognizes markers that separate adults with COPD from healthy subjects (de Laurentiis et al., 2008), unstable CF from stable CF (Montuschi et al., 2012), CF from PCD (Montuschi et al., 2014), and asthma from COPD (Maniscalco et al., 2018b). Furthermore, NMR-based metabolomics was able to demonstrate that the phenotype of obese asthmatic patients is fully different from that of patients independently affected by asthma or obesity (Maniscalco et al., 2017).

In the present study, we investigated the effects of the Pidotimod in non-cystic fibrosis bronchiectasis (NCFB) patients from EBC samples. By using NMR-based metabolomics, we aimed at uncovering the metabolites and related pathways affected by the immunostimulator, which may become useful indications of the immune response.

## Materials and Methods

### Patients

All patients were recruited from the Respiratory Department of the Monaldi Hospital (Naples, Italy). We enrolled 40 adults with proven and documented diagnosis of idiopathic or post-infectious bronchiectasis with HRCT affecting two or more lobes; history of at least 4 bronchial infections in the previous year characterized by fever and/or cough with increased sputum; stable pulmonary function with an FEV_1_ ≥ 80% of predicted; no asthma or COPD, stable drug treatment in the 4 weeks prior to screening; and ability to read and complete questionnaires. A second set (the test group, not considered for the primary analysis) included 20 NCFB patients and was used as a control set to verify blindly the models’ reliability. They were collected under similar clinical and experimental conditions. Pidotimod was taken at a dose of 800 mg b/d for 3 weeks a month for 6 consecutive months. From these patients, EBC was collected before Pidotimod administration and after 6 months from the first administration. The exacerbation was defined as worsening of two of the following signs or symptoms for at least two consecutive days, and requiring antibiotic treatment: dyspnea, wheezing, cough, amount of sputum, and sputum purulence with subsequent antibiotic treatment.

The local Ethical Committee approved the study, and an informed written consent was obtained from patients.

### Exhaled Breath Condensate Collection and Processing

All subjects were asked to refrain from food intake for 8 h before the test and from alcoholic drinks for 18 h before collecting the EBC, which they confirmed before sample collection. EBC was collected in random order and in the same room with a TURBO-DECCS condenser set at −5.0 ± 1.0°C (Medivac, Pilastrello, Parma, Italy, medivac.it). Briefly, all subjects were asked to breathe at tidal volume through the mouthpiece for 15 min while sitting comfortably and wearing a nose clip. They were instructed to seal their mouth around the mouthpiece, which was kept dry by periodically swallowing excess saliva. They were also asked to rinse their mouth thoroughly before and every 5 min during the test. We obtained, on average, 2.0 ± 0.3 ml (mean ± SD) of EBC from each subject. Volatile substances were removed by a gentle nitrogen gas flow for 3 min. EBC samples were immediately sealed in polypropylene tubes and stored first in dry ice and then at −80°C until NMR acquisition (de Laurentiis et al., 2008).

The salivary contamination of the samples was tested by measuring their α-amylase activity. The colorimetric reaction (Infinity Amylase Reagent, Sigma, Milan, Italy) is based on the hydrolysis of starch. The absorption was detected at 540 nm with a spectrophotometer (HP8453E Agilent Technologies Italia S.p.A., Cernusco sul Naviglio, Milan, Italy). The limit of detection was 0.078 U/ml. Saliva contamination was also checked with the 1D NMR spectra, in which contaminated spectra present signals from carbohydrates (which are absent in EBC spectra). Except for one sample from V_0_ (untreated) class and two samples from V_1_ (treated) group, which presented saliva contamination in both the amylase test and NMR spectra, the remaining samples (19 untreated and 18 treated) were uncontaminated. In fact, the intense saliva signals, originating from carbohydrates and resonating between 3.3 and 5.5 ppm (de Laurentiis et al., 2008), were absent in the EBC spectra. Therefore, the samples analyzed were 19 untreated control patients, and 18 were Pidotimod-treated. Regarding the external test group used to verify the model, we considered all of the 20 samples as none of them was contaminated by saliva.

The room temperature remained constant (24°C ± 1.0°C) throughout the sampling period. Possible air contaminants in the collecting room were monitored with a dedicated sampling pump for air monitoring (Zambelli EGO PLUS TT; Zambelli, Milan, Italy), working at a flow rate of 8 l/min and tidal volume (500 ml) into the condenser, so as to simulate human breath. The pump was connected to the condenser outlet for 15 minutes, and special filters (3M Particulate Filters P100; 3M Italia, Milan, Italy; tested against particles approximately 0.3 µm in size) for respiratory protection were applied to the one-way valve of the mouthpiece condenser used for the whole set of experiments. NMR spectra of condensed room air from the collecting device were devoid of signals, confirming the absence of air pollutants (data not shown).

To reduce the risk of contamination by inhaling hospital air, subjects were sampled after a 30-minute rest in the greenhouse of the Department of Respiratory Medicine, which was shown to be contaminant free as described above for the collecting room.

### NMR Sample Preparation

EBC samples were rapidly defrosted. To provide a field frequency lock, 70 ml of a ^2^H_2_O solution (containing 0.1 mmol/l sodium TSP as a chemical-shift reference for ^1^H spectra and sodium azide, a bacteriostatic agent at 3 mmol/l) was added to 630 ml of EBC, reaching 700 ml of total volume.

### NMR Spectroscopy Measurements

All spectra were recorded on a 600-MHz Bruker AVANCE-III Spectrometer (Bruker BioSpin GmbH, Rheinstetten, Germany) equipped with a CryoProbe. 1D ^1^H-NMR spectra were collected at 27°C with the excitation sculpting pulse sequence to suppress the water resonance. We used a double-pulsed field gradient echo, with a soft square pulse of 4 ms at the water resonance frequency and gradient pulses of 1 ms each in duration, adding 128 transients of 64k complex points, with an acquisition time of 4 s/transient. Time-domain data were all zero-filled to 128k complex points, and before Fourier transformation, an exponential multiplication of 0.6 Hz was applied.

2D clean TOCSY spectra were recorded by using a standard pulse sequence and incorporating the excitation sculpting sequence for water suppression (Griesinger et al., 1988). In general, 320 equally spaced evolution–time period t_1_ values were acquired, averaging 4 transients of 2,048 points. Time-domain data matrices were all zero-filled to 4,096 points in both dimensions, and before Fourier transformation, a Lorentz-to-Gauss window with different parameters was applied for both t_1_ and t_2_ dimensions for all the experiments. The spectral positions of the lines (“resonances”) in both homonuclear 1D and 2D spectra were referred to the spectral position of the signal originating from 0.10 mmol/l TSP, which was assumed to resonate at a δ value of 0.00 ppm.

For the natural abundance of 2D ^1^H-^13^C HSQC spectra, we used an echo-antiecho phase-sensitive pulse sequence by using adiabatic pulses for decoupling (Kay et al., 1992). Hundred twenty-eight equally spaced evolution–time period t_1_ values were acquired, averaging 48 transients of 2,048 points and using GARP4 for decoupling. The final data matrix was zero-filled to 4,096 in both dimensions and apodized before Fourier transformation by a shifted cosine window function in t_2_ and in t_1_. Linear prediction was also applied to extend the data to twice their length in t_1_. The spectral positions of the “resonances” were referred to the lactate signal (βCH_3_), which was assumed to resonate at 1.33 ppm for ^1^H and 20.76 ppm for ^13^C.

### Statistical Analysis

Since there are no standardized methods for evaluating the power of the analysis for projection methods such as orthogonal projections to latent structures (OPLS) analysis, we consider our study a pilot study for which no *a priori* power analysis was possible. Since biomarkers and their concentration changes that could determine class separation were unknown before analysis, we used our results to backward evaluate the power of our analysis.

By varying the parameters 1−α from 95 to 99.9% and 1−β from 80 to 99.9%, and using the accuracy percentages obtained for our validation tests for untreated/treated patients, for a 1−α value of 95% and a 1−β value of 80%, we obtained a number of subjects corresponding to 14 ± 2 for both classes, whereas for a 1−α value of 99.9% and a 1−β value of 99.9%, we obtained a number of 16 ± 2 for untreated/treated patients.

Here, we analyzed 19 untreated and 18 treated patients, corresponding to a number of enrolled subjects that is in line with the backward analysis. Typically, the 1−α value is 95%, and the 1−β value is at least equal to 80%, whereas a value of 99.9% represents an extreme requirement. On the other hand, the permutation and the validation tests done within the OPLS Discriminant Analysis (OPLS-DA) have confirmed the existence and validity of the model and avoided the overfitting problem (see below).

**Figure 1:**
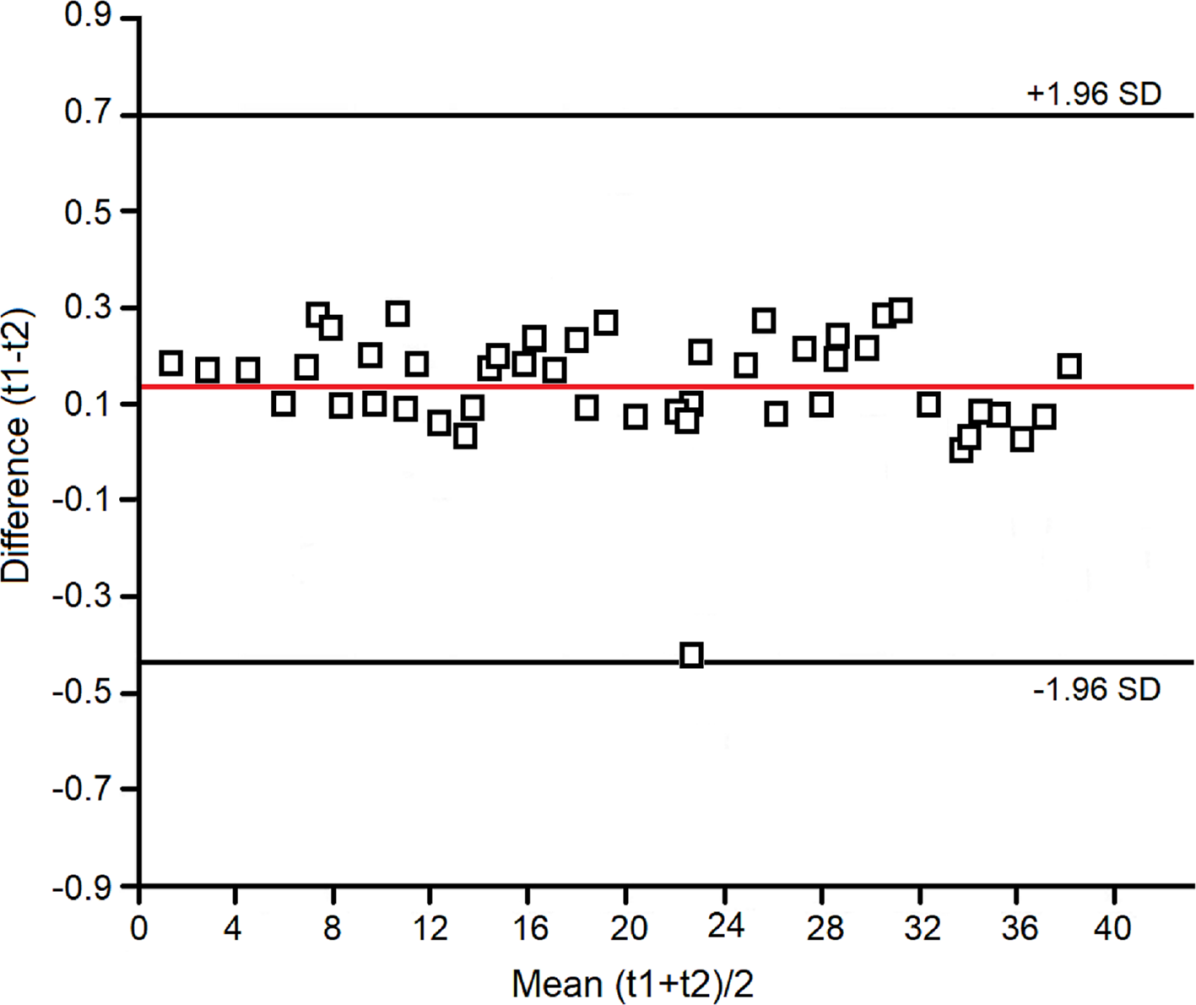
Bland–Altman plot for the spectral zones considered in the evaluation of within-day repeatability of the NMR measurements (see *Materials and Methods*).

Within-day, between-day, and technical repeatability, and detection limits were assessed as previously reported (Carraro et al., 2007; Montuschi et al., 2012; de Laurentiis et al., 2013). Assessment of within-day repeatability of NMR spectra was according to Bland and Altman, and all spectral lines were considered. Two EBC samples were collected twice within the same day (at times 0 h and 12 h) from four untreated and four treated subjects. Each spectrum was subdivided in six equally corresponding regions, while the region 5.20–4.50 ppm, containing the residual water resonance, was excluded. All regions were integrated and normalized to the total spectrum area. We obtained 6 parameters (the integrated fractional regions) for each spectrum, which for 8 subjects amounted to 48 values. All values except one fell within the 2SD range, indicating a good within-day repeatability (**Figure 1**).

Between-day repeatability was expressed as ICC of the 4.4–0.4-ppm spectral region. The ICC was 0.99. Technical repeatability was assessed by repeating NMR spectroscopy on four different samples (two from untreated and two from treated patients) four times consecutively. The ICC for the 4.4–0.4-ppm spectral region was 0.98. The measured detection limit was 0.07 ± 0.02 μM for phenylalanine signals (not shown), the lowest detectable concentration with respect to the internal TSP concentration standard.

We used multivariate analysis to discriminate signals/metabolites and identify hidden phenomena and trends in ensembles of spectra. Proton NMR spectra were automatically data reduced to 390 integral segments (“buckets”), each of 0.02 ppm, using the AMIX 3.6 software package (Bruker BioSpin GmbH, Rheinstetten, Germany), within the 0.10–8.60-ppm region. The residual water resonance region (5.20–4.50 ppm) was excluded, and each integrated region was normalized to the total spectrum area to avoid possible dilution effects on the signals.

The data format obtained (X matrix) was imported into SIMCA-P +14 package (Umetrics, Umeå, Sweden), and principal components analysis (PCA) and OPLS-DA were performed. PCA is an unsupervised technique that reduces dimensionality without *a priori* knowledge of sample categories, while OPLS-DA is a supervised technique and requires *a priori* knowledge of sample categories. Mean-centering was applied as data pre-treatment for PCA, while Pareto scaling was used prior to OPLS-DA. PCA was performed to reduce the dimensionality of the data and to reveal any clustering of the four study groups in an unsupervised manner. Once class homogeneity was assessed for each group, supervised OPLS-DA was applied, where a dummy variable Y matrix was used. Supervised regressions were conducted comparing two groups at a time in order to generate predictive models. The model quality was evaluated by using the goodness-of-fit parameter (R^2^) and the goodness-of-prediction parameter (Q^2^). We actually tested both OPLS and orthogonal signal correction (OSC) routines together with the PLS-DA to verify data fitting and possible data over-fitting, which was excluded. The obtained OPLS models turned out to be improved in terms of both predictive and interpretive abilities.

Associated scores plots were used to visualize sample class distribution and highlight putative markers for classification. To check for model overfitting and data regression performance, each OPLS-DA model was validated by an internal iterative 7-round cross-validation, response to permutation test (800 repeats), and analysis of variance testing of cross-validated predictive residuals (CV-ANOVA). Statistical significance for selected metabolites and PFT was determined by parametric (Student’s t) or non-parametric (Wilcoxon) tests for paired data, according to the results of normality test performed to evaluate each distribution (Shapiro–Wilk test). Chi square was used for comparing proportions. For all tests, only *p* values < 0.05 were considered as statistically significant.

## Results

**Figure 2:**
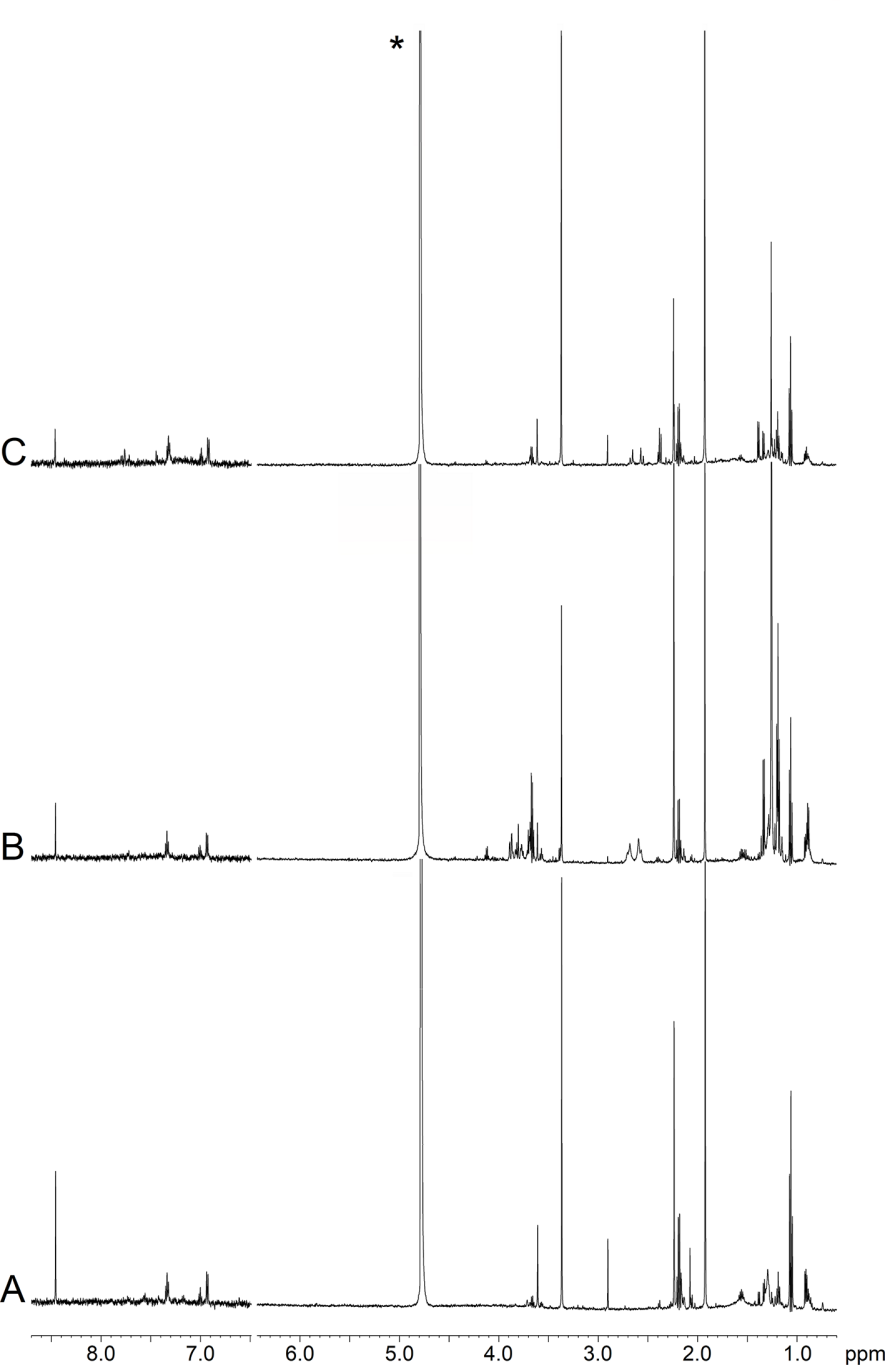
Representative ^1^H-NMR spectra of EBC samples. **(A)** Healthy subject (HS); **(B)** non-cystic fibrosis bronchiectatic (NCFB) patient; **(C)** NCFB patient treated with Pidotimod. All signals were assigned to single metabolites by resorting to 2D NMR experiments and referring to published data on metabolite chemical shifts. Absorption (related to the intensity) is plotted on the y-axis, and magnetic field strength is plotted on the x-axis. The 8.7−6.5-ppm region has been vertically expanded 32 times. The asterisk in spectrum **(C)** marks the residual water resonance.

**Figure 2** depicts representative NMR profiles (spectra) of EBC samples from a NCFB patient before (B) and after (C) Pidotimod treatment. As a comparison, the spectrum of a healthy subject is also reported (A). Spectral lines were assigned to specific metabolites by resorting to 2D correlation experiments (not shown; see *Materials and Methods*) with the aid of published chemical-shift data on metabolites, and sample spiking with corresponding standards. A qualitative evaluation of the spectra suggests that the Pidotimod treatment (**Figure 2C**) simplifies the EBC profile with respect to the untreated NCFB (the group of the signals between 4.0 and 3.5 ppm, the group around 1.0 ppm as well as the intensity of some sharp singlets in spectrum B), becoming comparable to the healthy subject profile (spectrum 2A). However, some peculiarities are still present. For example, compared with the NCFB profile (2B), the treated NCFB (2C) maintains the citrate and lactate signals at 2.51 and 1.33 ppm, respectively, although reduced in intensity. This would indicate that the Pidotimod treatment affects the NCFB metabolic phenotype (“metabotype”) generating a different metabotype that relates to (but is not the same than) the normal one. That is, Pidotimod administration does not heal from NCFB but improves the pathophysiological status (*vide infra*).

To obtain relevant biochemical information from NMR data, each spectrum was analyzed trough multivariate statistical analysis, namely, PCA and OPLS-DA.

**Figure 3:**
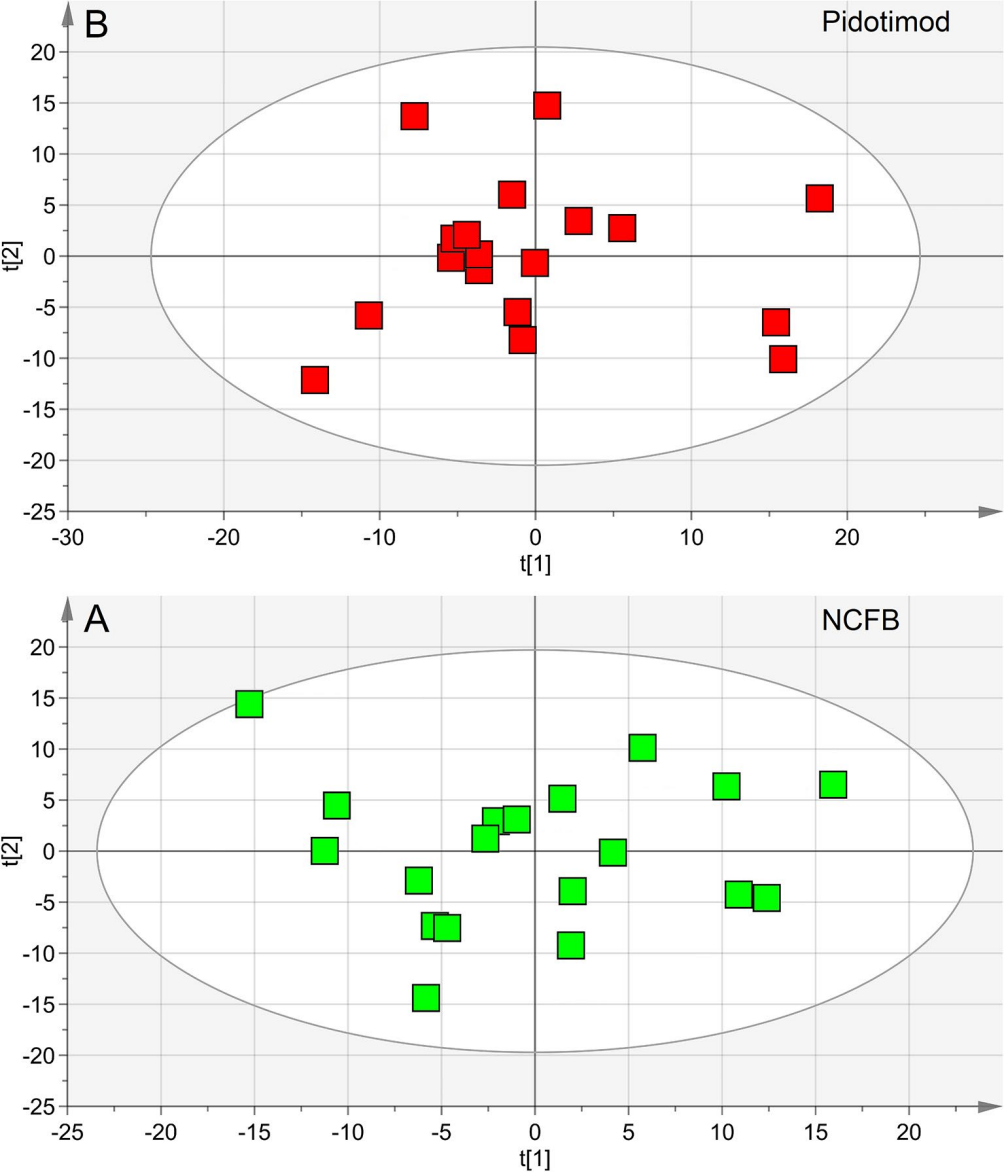
Principal component analysis (PCA) of EBC samples. **(A)** Scores plot relative to NCFB patients before treatment (green squares); **(B)** scores plot for the same patients after Pidotimod treatment (red squares). No satisfactory classification model was obtained, showing **(A)** R^2^ = 0.33 and Q^2^ = 0.16, and **(B)** R^2^ = 0.29 and Q^2^ = 0.21. The labels t[1] and t[2] along the axes represent the scores (the first two partial least squares components) of the model.

We first verified the homogeneity of all classes (V_0_ and V_1_ patients) and the presence of possible outliers by applying PCA before the application of supervised analysis. **Figure 3** reports the PCA scores plots obtained for the EBC samples collected from the NCFB before (3A, green squares) and after (3B, red squares) Pidotimod administration. No discernible patterns were identified, neither subgroups nor outliers, confirming that the classes are homogeneous. Therefore, all of the NMR spectra from the 19 untreated and the 18 treated samples were included in the statistical analysis. The model quality was evaluated by using the goodness-of-fit parameter (R^2^) and the goodness-of-prediction parameter (Q^2^). For them, acceptable values must be ≥0.5, with |R^2^−Q^2^| < 0.2–0.3. For the depicted models, we obtained two principal components with R^2^ = 0.33 and Q^2^ = 0.16, and R^2^ = 0.29 and Q^2^ = 0.21 for plots 3A and 3B, respectively, which is an indication of the absence of subgroups. The existence of probable outliers was also verified in the external test set of 20 NCFB patients. PCA analysis detected neither subgroups nor outliers, with quality parameters R^2^ = 0.24 and Q^2^ = 0.19, confirming class homogeneity (not shown).

**Figure 4:**
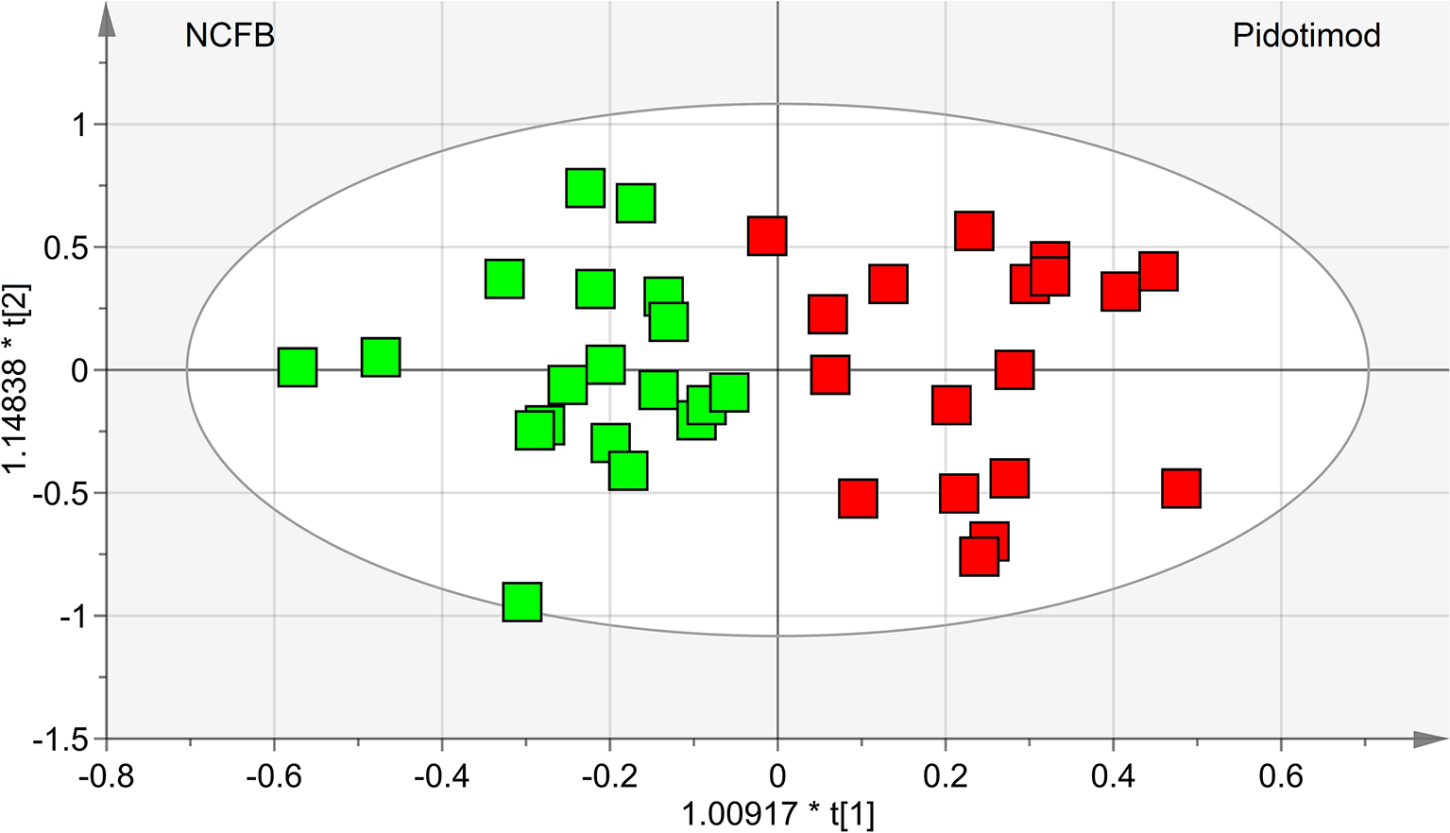
OPLS-DA of EBC samples. Scores plot (97%, *p* < 0.0001) showing the degree of separation of the model between NCFB patients before (green squares) and after Pidotimod treatment (red squares). For this model, we recorded R^2^ = 0.86 and Q^2^ = 0.79. The labels t[1] and t[2] along the axes represent the scores (the first two partial least-squares components) of the model, which are sufficient to build a satisfactory classification model.

**Figure 5:**
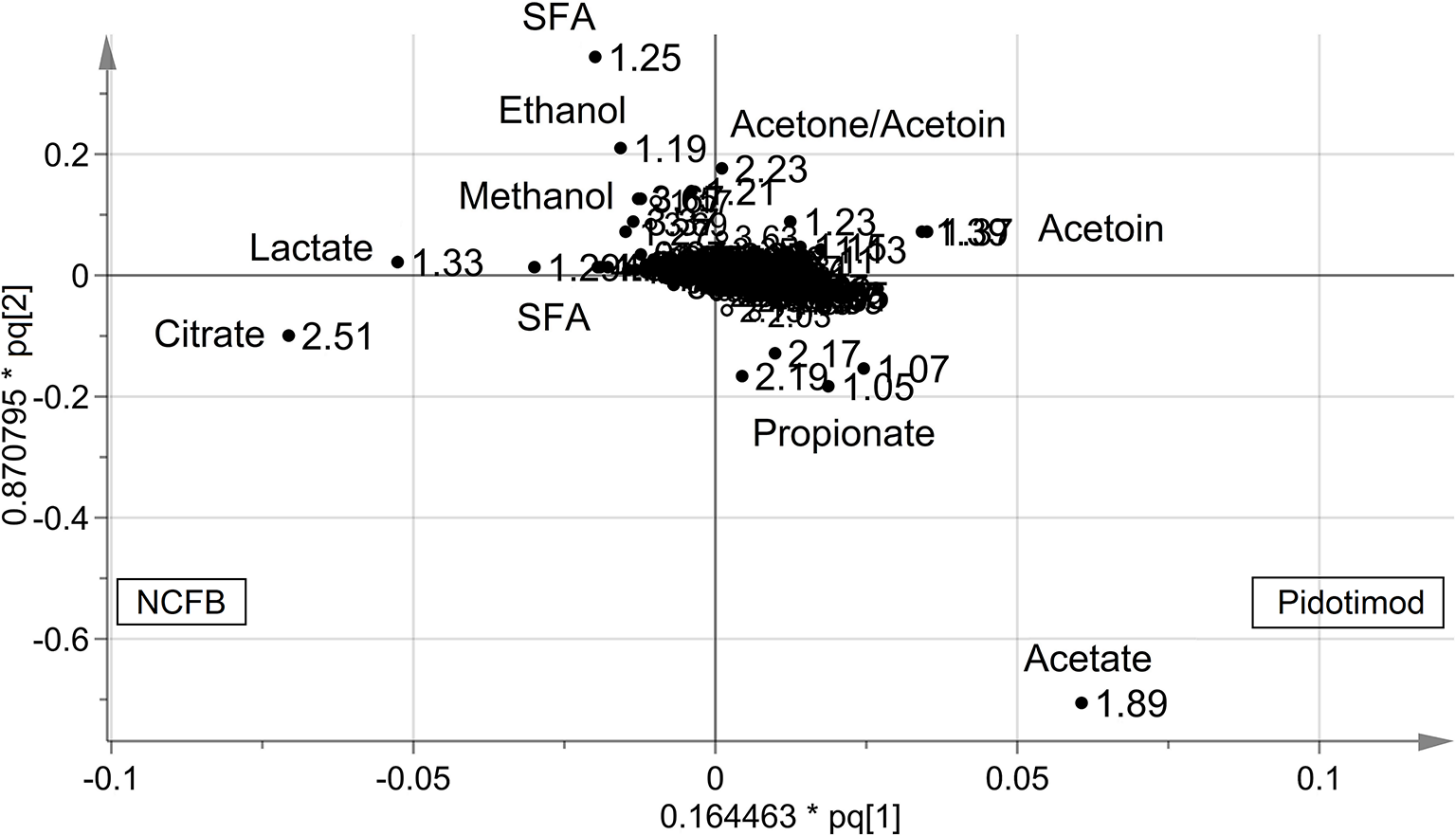
Loadings plot associated with the OPLS-DA analysis reported in **Figure 4**. Metabolites responsible for between-class differences are labeled. Numbers refer to buckets’ chemical shifts (spectral positions), and the discriminating metabolites are explicitly labeled. The pq[1] and pq[2] values refer to the weight that combines the X and Y loadings (p and q).

OPLS-DA was next performed, obtaining a strong regression model (97%, *p* < 0.0001; **Figure 4**) between NCFB patients before (green squares) and after (red squares) Pidotimod administration. The resulting supervised model was tested by iteratively predicting the class membership of every sample, and the results were used to evaluate R^2^ and Q^2^. For this model, we recorded R^2^ = 0.86 and Q^2^ = 0.79. From the associated loadings plot (**Figure 5**), we identified the NMR signals (*i.e.*, the metabolites) responsible for the class separation. Namely, 3.37 ppm (methanol), 2.51 ppm (citrate), 2.23 ppm (acetone/acetoin), 2.19 and 1.07 ppm (propionate), 1.89 ppm (acetate), 1.37 ppm (acetoin), 1.33 ppm (lactate), 1.29 and 1.25 ppm (saturated fatty acids, SFA), and 1.19 ppm (ethanol). The signals on the left side (like 2.51 and 1.33 ppm) were more expressed in the NCFB V_0_ class, while those on the right side (like 1.89 and 1.37 ppm) were increased in the Pidotimod V_1_ class. In addition, the signals at 1.89, 1.25, and 1.19 ppm were more responsible for intra-class differences than for inter-class differences, because their contribution is larger along the orthogonal component than the parallel or predictive component.

**Figure 6:**
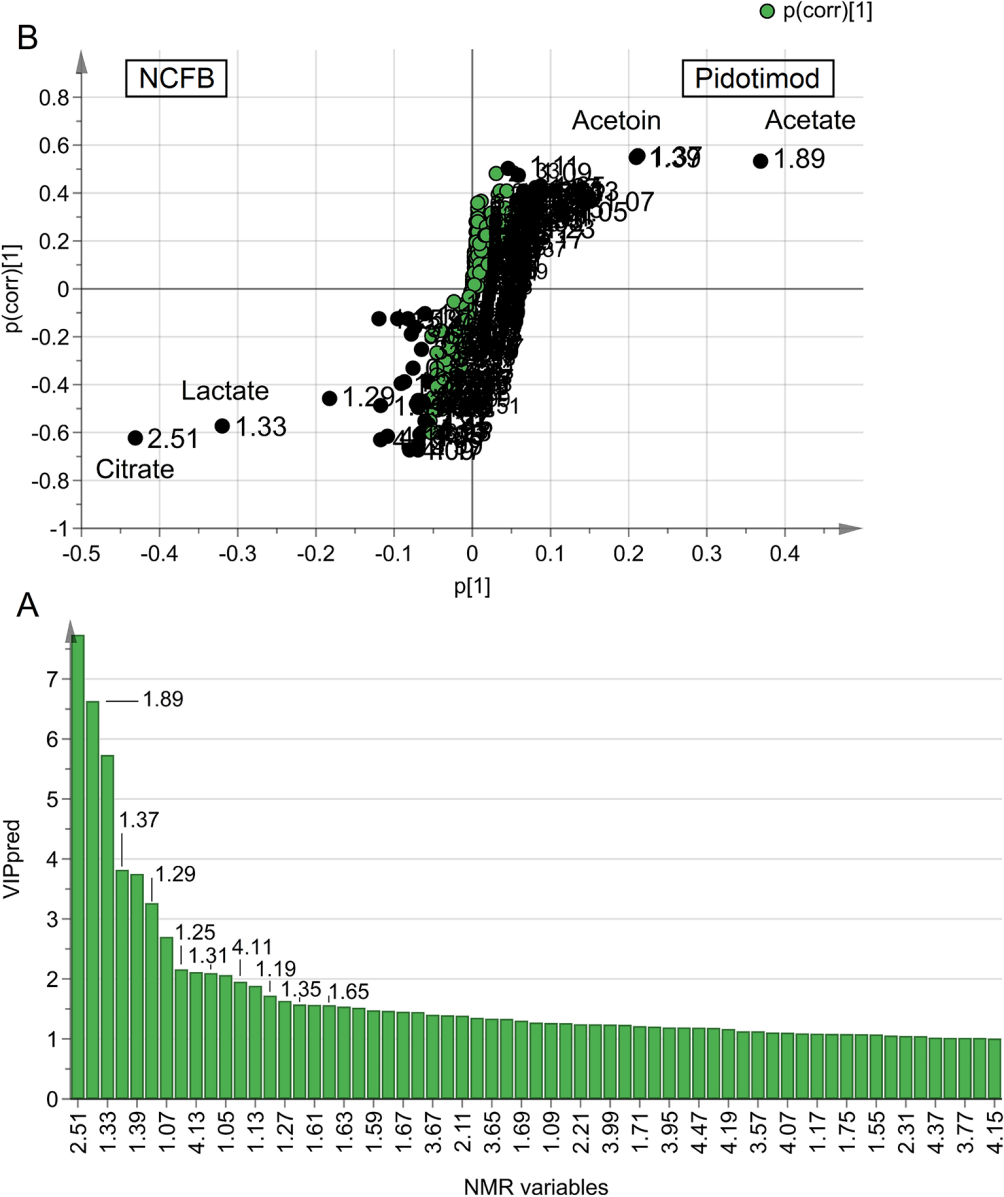
Variables of importance plot (VIP) and S-plot associated with the OPLS-DA analysis reported in **Figure 4**. **(A)** VIP reporting on the x-axis the buckets [identified with chemical shift, in ppm, and labeled “NMR variables” (variable identity)], while on the y-axis, labeled “VIP_pred_,” the strength of the labeled metabolites in the classification between patient classes is reported. Error bars represent 95% CIs. **(B)** S-plot reporting p(corr) against the predictive loading vector p of the model.

**Figure 7:**
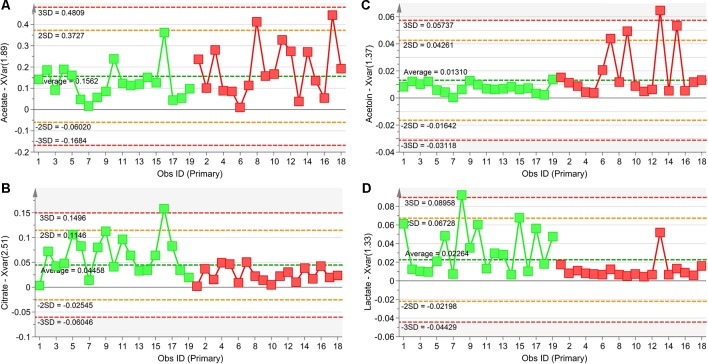
Plots of the NMR variables values for the four statistically significant metabolites contributing to the separation of NCFB groups. Selected bucket variations are scaled to the total spectral area and correspond to acetate (1.89 ppm, panel **A**), citrate (2.51 ppm, panel **B**), acetoin (1.37 ppm, panel **C**), and lactate (1.33 ppm, panel **D**). Patients before Pidotimod treatment are identified by green squares, while red squares identify patients after administration. Numbers on the x-axis refer to EBC samples used for the analysis; the y-axis reports the bucket variation. Pidotimod increases the levels of acetate and acetoin (**A** and **C** panels) and reduces the levels of citrate and lactate (**B** and **D** panels).

The statistically significant metabolites are depicted in the Variable Importance in the Projection (VIP) plot (**Figure 6A**) and in the corresponding S plot (**Figure 6B**). It is important to notice that when more than one chemical group belonging to the same metabolite is observed in the NMR spectrum, the corresponding buckets are all reported in the VIP plot. For example, the two vertical bars (“buckets”) in the VIP at 1.33 and 4.11 ppm both originate from lactate, being the signals of the methyl and methine protons, respectively. Considering VIP > 1 and *p*_corr_ > 0.5, we identified four statistically significant variables/metabolites: 2.51 ppm (citrate), 1.89 ppm (acetate), 1.37 ppm (acetoin), and 1.33 ppm (lactate), whose variations are reported in **Figure 7**. The four statistically relevant metabolites were then used for between-group classification. We confirmed the above model obtaining only a 4% reduction of the quality parameters (95%; R^2^ = 0.81; Q^2^ = 0.72; *p* < 0.0008). In particular, acetate and acetoin concentration increased in V_1_ class (after Pidotimod administration), while citrate and lactate decreased. Therefore, these metabolites are relevant biomarkers with an important role in the physiological answer to Pidotimod treatment.

**Figure 8:**
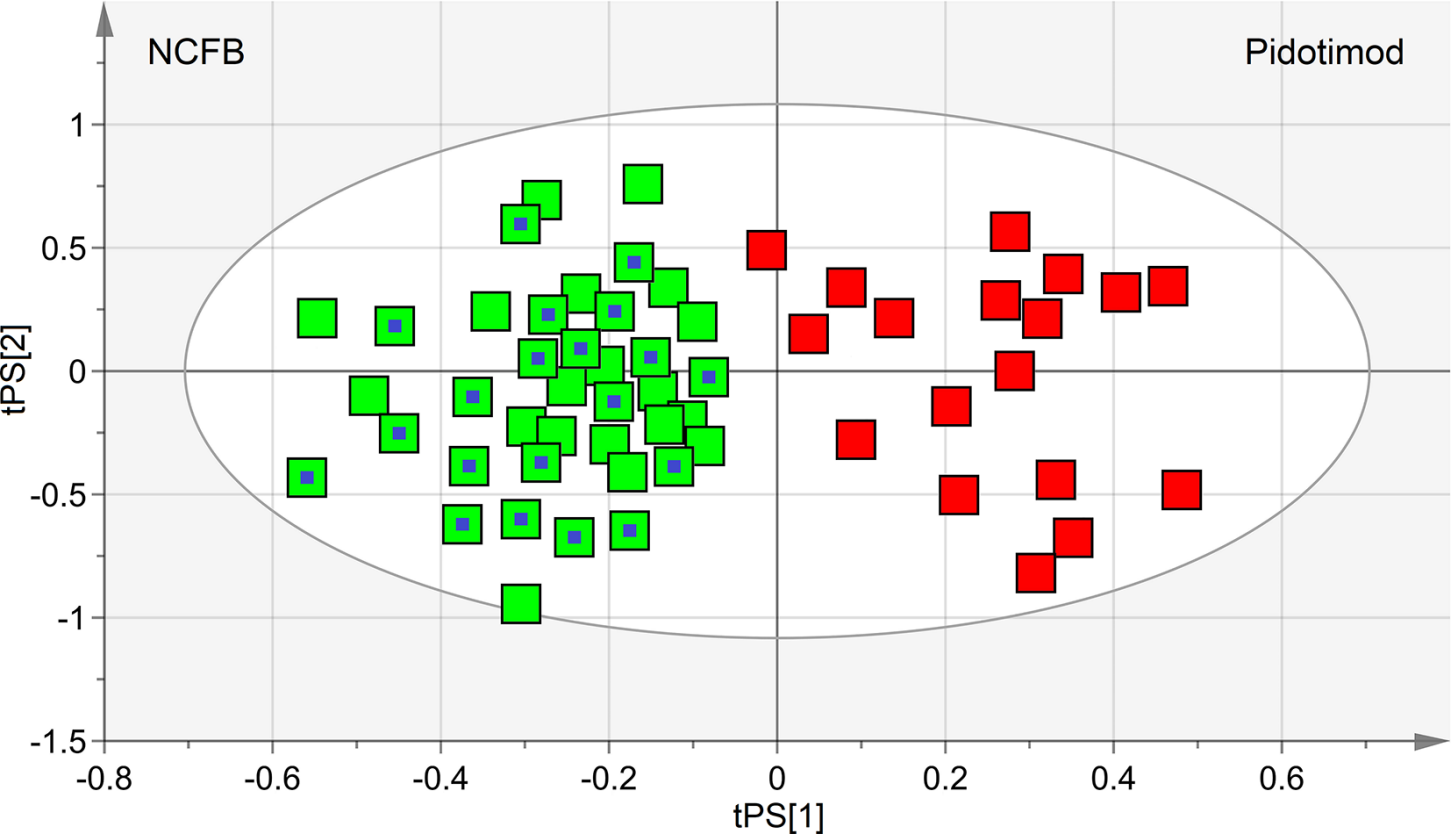
Predictive scores plot of the validation untreated NCFB set of samples. Untreated NCFB samples and Pidotimod-treated samples of the training set are indicated by green squares and red squares, respectively, while the predicted set of 20 new untreated NCFB samples is represented as green squares with a central blue dot. The position of each sample is identified by the associated predictive coordinates (tPS).

The performance of the OPLS-DA model was also evaluated using a sample set not included in the model calculation. Specifically, we tested an external data set of EBC samples obtained from 20 NCFB patients not included in the primary analysis and collected under similar experimental conditions. They were projected onto the corresponding statistical model, and the results are displayed in **Figure 8**. We obtained high-quality parameters (R^2^ = 0.87 and Q^2^ = 0.85) as all samples (blue-dotted green squares) are correctly located in the predicted NCFB area. The discriminating metabolites characterizing the NCFB-Pidotimod coincide with those found for the above calculated model.

**Figure 9:**
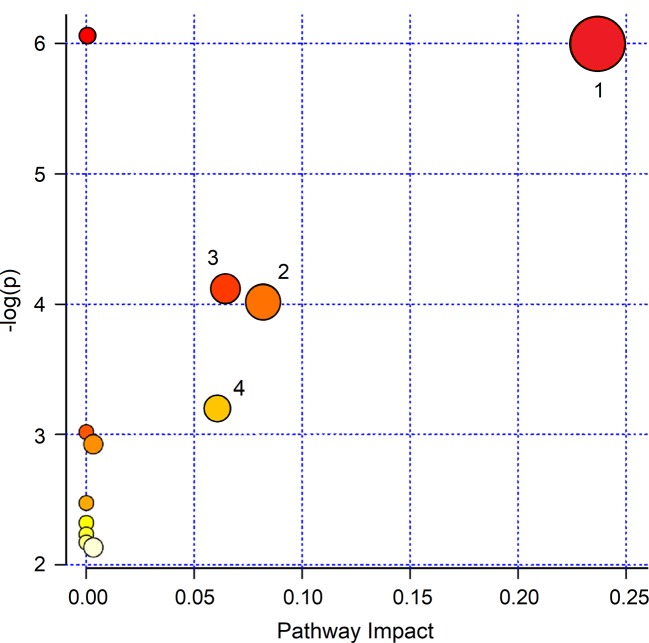
MetaboAnalyst 4.0 pathway impact based on metabolites responsible for the class separation (see *Materials and Methods* section). Circles represent all metabolic pathways potentially involved in class separation. Numbers identify the metabolisms showing the highest impact: 1, pyruvate; 2, citrate; 3, sulfur; 4, methane. They present different *p* values and impact parameters (see *Results* section).

To interpret the biological relevance of the data, using the MetaboAnalyst 4.0 platform (Chong et al., 2018), we examined the metabolic pathways in which the differently regulated metabolites are involved. The found pathways are depicted in **Figure 9**, which reports the impact of each pathway *versus* *p* values. Using the discriminating metabolites, we uncovered 12 metabolic pathways that appear to be significantly dysregulated upon treatment. From them, we extrapolated pyruvate (*p* = 1.00×10^−6^; impact, 0.24), citrate (*p* = 9.77×10^−5^; impact, 0.08), sulfur (*p* = 7.94×10^−5^; impact, 0.065), and methane (*p* = 6.31×10^−4^; impact, 0.06) metabolisms as the most probable. They might contain molecular species potentially relevant as biomarkers for specific pathophysiologic processes within the respiratory metabolome.

No difference was observed in FVC, and FEV_1_ in patients who had taken Pidotimod (FEV_1_ from 2.4 ± 0.9 to 2.8 ± 0.6 l and FVC from 2.9 ± 1.1 to 3.2 ± 1.1 l) as compared to controls (FEV_1_ 2.6 ± 0.9 to 2.5 ± 0.9 l and FVC 2.8 ± 0.9 to 3.1 ± 0.9 l), *p* always > 0.05, although the number of exacerbations was significantly lower (7 in treated group *vs.* 20 in untreated group, *p* < 0.001).

**Figure 10:**
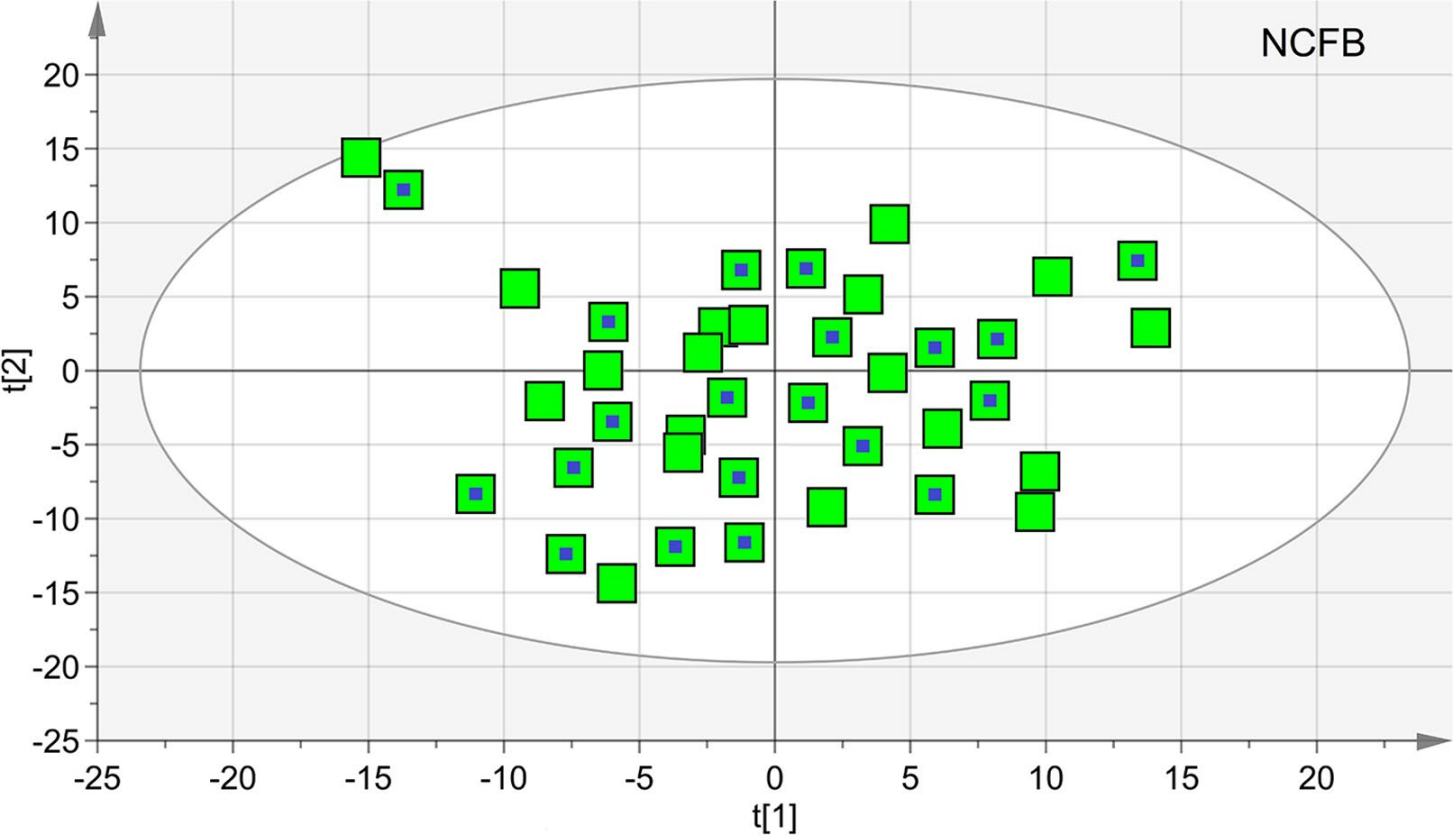
Principal component analysis (PCA) of EBC samples of the controls at V_0_ and V_1_ times. The scores plot included 39 samples, 19 at V_0_ (green squares), and 20 collected after 6 months (green squares with a central blue dot). None of the latter presented saliva contamination, and therefore, all of the initial 20 NCFB patients were included in the analysis. The quality parameters were R^2^ = 0.27 and Q^2^ = 0.21, which are an indication of the absence of subgroups.

The untreated samples were also examined after 6 months (V_1_). The comparison of the NMR spectra and the (unsupervised) PCA and (supervised) OPLS-DA analyses did not show relevant changes. In particular, we verified the homogeneity of samples and the presence of possible outliers by applying PCA. The PCA scores plot achieved for the class including the control samples. In particular, at the 19 samples collected at V_0_, we added 20 samples obtained after 6 months. Since none of the 20 selected NCFB patients presented saliva contamination, they were all included in the analysis and amounted to 39 samples. No discernible patterns, neither subgroups nor outliers were identified (**Figure 10**), confirming that they formed a homogeneous class, without notable differences in the metabolic profiles after 6 months. The goodness-of-fit parameter (R^2^) and the goodness-of-prediction parameter (Q^2^). The quality parameters we obtained were R^2^ = 0.27 and Q^2^ = 0.21, which are an indication of the absence of subgroups.

We next applied OPLS-DA obtaining no regression model (32%, *p* < 0.19) between NCFB controls at 0 time and after 6 months. For this model, we recorded R^2^ = 0.36 and Q^2^ = 0.29, confirming that no relevant metabolic differences were observed after 6 months in the controls.

We found no correlation between metabolomic data and spirometric results in the study group. Identically, no correlations were observed between metabolites and any of the anthropometric parameters (listed in **Table 1**).

**Table 1:** Patient characteristics^a^.

Anthropometric data	Controls	Pidotimod	All	Validation set
n	19	18	37	20
Female gender	60%	70%	65%	68%
Age (years)	56 ± 12	57 ± 13	56.7 ± 16	55.9 ± 14
BMI (kg/m^2^)	25.3 ± 4.2	28.5 ± 4.8	27.5 ± 4.9	26.2 ± 4.7
FEV_1_ pre-BD (l)	2.6 ± 0.9	2.4 ± 0.9	2.5 ± 0.9	2.4 ± 0.8
FVC pre-BD (l)	3.1 ± 0.9	2.9 ± 1.1	3.0 ± 1.0	3.0 ± 0.9
FEV_1_/FVC	84 ± 8.2	85 ± 8.0	85 ± 7.9	83 ± 8.3
SGRQ	45.8 ± 1.6	47.8 ± 1.8	46.8 ± 1.3	44.6 ± 1.9
Exacerbations in previous year	4 ± 2	4 ± 2	4 ± 2	4 ± 2

a Data are expressed as numbers or means ± SDs. One-way ANOVA and unpaired t tests were used for comparing groups. Significance was defined as a p ≤0.05. Mean ± standard deviation or percentage.

FEV^1^ pre-BD, forced expiratory volume at 1 second pre-bronchodilator; FVC pre-BD, forced vital capacity pre-bronchodilator; SGRQ, San George Respiratory Questionnaire.

## Discussion

In our study, NMR-based metabolomics of EBC has shown that the immunomodulatory Pidotimod affects the respiratory metabolic phenotype (“metabotype”) of NCFB patients. Pidotimod was administered according to clinical practice, at a dose of 800 mg b/d for 3 weeks (21 days). At such dose, Pidotimod induces dendritic cells (DCs) maturation, up-regulates the expression of HLA-DR and of co-stimulatory molecules, stimulates DCs to release pro-inflammatory molecules driving T-cell proliferation and differentiation toward a Th1 phenotype, enhances natural killer (NK) cells functions, and promotes phagocytosis (Esposito et al., 2015), resulting in a significant upregulation of both innate and, possibly, adaptive immune responses (Trabattoni et al., 2017).

In our respiratory metabolic phenotype, the treatment altered the levels of methanol, citrate, acetone/acetoin, propionate, acetate, lactate, saturated fatty acids and ethanol, with citrate, acetate, acetoin, and lactate being statistically significant. In particular, after treatment, acetate and acetoin increased while citrate and lactate decreased. Pathway topology analysis indicated (in order of impact percentage) pyruvate, citrate, sulfur, and methane metabolisms as the most probable pathways affected by Pidotimod.

Pyruvate is the end product of glycolysis, which finally enters the mitochondria where it sustains the citric acid cycle. Disorder in pyruvate metabolism is involved in several diseases, including COPD (Gray et al., 2014), in which removal of pyruvate dysmetabolism improved physical performance, which is an important therapeutic goal in COPD (Calvert et al., 2008). Under aerobic conditions, pyruvate enters the tricarboxylic acid (TCA) cycle to produce also citrate. Consumption of exogenous citrate blocked growth and increased apoptosis in a mesothelioma cell line highly resistant to cisplatin (Zhang et al., 2009) and inhibited proliferation of A549 lung cancer cells *in vitro* and *in vivo* (Hanai et al., 2012). Such a potential antitumor function for citrate is attributed to an effect on immune response and signal transduction pathways (Ren et al., 2017). We found that, upon Pidotimod treatment, the level of citrate decreased together with lactate. Therefore, it is conceivable that the immunomodulator activates citrate consumption in bronchiectatic patients, as a part of the general immune response. It is suggested that citrate could become a biomarker to monitor the response to the Pidotimod therapy *via* NMR-based metabolomics of EBC.

The lactate decrease in treated NCFB patients could have a direct link with reduced citrate level. In red blood cells incubated with deuterated citrate, *ca.* one-third of the lactate that could not be explained by glucose oxidation and 2,3‐diphosphoglycerate consumption alone originates from citrate uptake and metabolism (D’Alessandro et al., 2017). Recent studies suggest that lactate can be a source of carbon for the citric acid cycle (Hui et al., 2017). As such, the parallel reduction of citrate and lactate in treated NCFB patients could be part of the immune activation brought about by Pidotimod, in which citrate and lactate are both involved *via* the citric acid cycle.

Sulfur-containing metabolites play an important role in maintaining and supporting immune functions by modulating the actions of oxidant stress in transcription factor activation (Grimble, 2006). For example, in animals under protein-deficient diet and showing inflammation after endotoxin injection, addition of methionine to the diet normalized tissue glutathione (GSH) content and upregulated lung neutrophils (Hunter and Grimble, 1994). Therefore, activation of the sulfur metabolism is part of the defense mechanism, which should favor a reduction of lung inflammation.

Methane metabolism is involved in the global carbon cycle, and, among the three types of known methanogenic pathways, the one converting acetate to methane [KEGG module M00357, www.genome.jp/kegg/ relates to the increased acetate found in treated NCFB patients. Methane metabolism is tightly connected with several metabolic cycles, including sulfur and pyruvate [KEGG map 00680, www.genome.jp/kegg]. It is also involved in the production of cellular energy, which suggests, together with the upregulation of the citric acid cycle, that the energy requirement is increased after treatment, recalling the improved exercise tolerance observed in COPD upon the upregulation of pyruvate metabolism (Calvert et al., 2008).

Acetate and acetoin increased after Pidotimod treatment. Acetate, a short-chain fatty acid, controls some proinflammatory mediators (Arpaia, 2014; Ishiguro et al., 2014) and drives leukocyte migration to inflammatory sites (Vinolo et al., 2011). Therefore, increased levels of acetate after Pidotimod administration responded to the drug immunomodulator activity.

Acetoin (3-hydroxy-2-butanone), which is detected in the breath of patients with cystic fibrosis, causes direct structural damage and likely interacts with the host immune system (Whiteson et al., 2014). It is the product of the detoxication process of acetaldehyde (Otsuka et al., 1996) but may also be a bacterial fermentation product produced by both pathogenic and non-pathogenic bacteria (Filipiak et al., 2012). The relative contribution of lung microbiome to the production of the acetoin found in the EBC samples is still unknown. In addition, reactive carbonyls such as diacetyl (2,3-butanedione) are contained in tobacco and many food (including butter), and consumer products are detoxified by carbonyl reductases in the lung, in particular, dicarbonyl/l-xylulose reductase (DCXR), a multifunctional lysosomal enzyme expressed in the airway epithelium. The reactive α-dicarbonyl group in diacetyl causes protein damage *in vitro*. DCXR metabolizes diacetyl to acetoin, its reduced product that lacks this α-dicarbonyl group (Ebert et al., 2015). Therefore, acetoin increases linked to Pidotimod treatment might be explained by the requirement of limiting pulmonary inflammation by activating the detoxification process of diacetyl groups stimulated by Pidotimod.

We are aware that the present study has some limitations. First, it is a non-controlled study and relies on a restricted number of subjects; therefore, our data are preliminary, and this should be considered a pilot study. Further studies on the effectiveness of Pidotimod in the treatment of NCFB patients are needed to determine prospectively the relevance of the perturbed metabolic pathways. However, we confirmed that NMR-based metabolomics is a noninvasive approach for the evaluation of therapy in respiratory diseases, with important implications for follow-ups and personalized treatments.

The metabolic changes are not immediately visible in systemic variations, and therefore, a direct link between clinics and metabolomics may not be manifested. Lack of correlation of metabolic data with clinical parameters could be related to the use of a single biomatrix (EBC) in the understanding of a complex system, which is globally represented by the clinical parameters (Maniscalco and Motta, 2017). The fact that the clinical indications do not always refer to molecules with a MW <2,000 Da may also support the absence of correlation. We are presently applying NMR-based pharmacometabolomics to multiple biofluids (serum, urine, EBC, and saliva) to obtain possible global parameters that could be compared with clinical parameters. By integrating the metabolomics profiles of different biofluids, we aim at better defining the pathways affected by immunomodulators.

Finally, the response to Pidotimod should have been better monitored by clinical parameters—for example, following the decrease of the pulmonary exacerbation occurrence. However, although the clinical parameters are central, we believe that our analysis may be complementary in the assessment of the Pidotimod effects, and that the evolution of pulmonary exacerbations can be efficaciously monitored *via* molecular determinants and altered pathways.

Considering these limitations, our findings, obtained by using an unbiased methodology, unequivocally indicate that NCFB patients when treated with Pidotimod present specific metabolic alteration that might be useful to follow the treatment. This possibility adds strong relevance to the use of EBC- and NMR-based metabolomics to the clinics of respiratory pathologies. The described statistical model defines the change in “metabotype” obtained after Pidotimod treatment. This is very important since NMR profiling makes no *a priori* assumptions about EBC components that may be associated with a particular therapy. Furthermore, it analyzes the sample in a multiparametric way, identifying new and unsuspected links between processes and pathways perturbed in a disease state.

## Data Availability Statement

The raw data supporting the conclusions of this manuscript will be made available by the authors, without undue reservation, to any qualified researcher.

## Ethics Statement

Protocol n. 5/18 OSS Maugeri IRCCS, Telese (BN). Committee of the Scientific Institute Pascale, Naples, Italy (23 May 2018).

## Author Contributions

MD, AMol, MM, and AMot ideated the study, enrolled patients, and discussed the study. DP, PC, AF, NS, and LP acquired the data and followed patients. DP, PC, and AMot performed statistical analysis. MD, AMol, MM, and AMot coordinated and discussed the study.

## Conflict of Interest

The authors declare that the research was conducted in the absence of any commercial or financial relationships that could be construed as a potential conflict of interest.
